# A METHODOLOGICAL APPROACH TO IDENTIFY EPISODES OF LUCIDITY AMONG PEOPLE WITH ALZHEIMER'S DISEASE

**DOI:** 10.1093/geroni/igac059.352

**Published:** 2022-12-20

**Authors:** Kyungmin Kim, Theresa Frangiosa, Virginia Biggar, Dawne Finnie, Lauren Bangerter, Joseph Gaugler, Maria Lapid, Joan Griffin

**Affiliations:** Seoul National University, Seoul, Seoul-t'ukpyolsi, Republic of Korea; UsAgainstAlzheimer's, Washington, District of Columbia, United States; UsAgainstAlzheimer's, Washington, District of Columbia, United States; Mayo Clinic, Rochester, Minnesota, United States; United Health Group, Edina, Minnesota, United States; University of Minnesota, Minneapolis, Minnesota, United States; Mayo Clinic, Rochester, Minnesota, United States; Mayo Clinic, Rochester, Minnesota, United States

## Abstract

People with late-stage Alzheimer’s disease and related dementias (ADRD) who are assumed to have lost coherent cognitive capacity may exhibit unexpected episodes of lucidity (EL). Given the transient nature and lack of scientific explanation of the phenomenon, EL is under-investigated and poorly understood. To better understand this phenomenon, we set out to develop an operational definition of EL. Based on survey data from former and current family caregivers participating in UsAgainstAlzheimer’s A-LIST® (N = 480), we defined four EL typologies. We then interviewed 25 caregiver respondents about their experiences and used analyzed qualitative data to refine the preliminary typologies. Finally, we conducted a Delphi consensus panel with clinicians, researchers, and health care providers with medical, pharmacological, and clinical expertise to describe potential explanations for EL to help further refine the typologies. Next we will test the validity of these typologies in a prospective, demographically diverse sample of current family caregivers.

